# Right ventricular myxoma obstructing the right ventricular outflow tract: a case report

**DOI:** 10.1186/1752-1947-8-435

**Published:** 2014-12-17

**Authors:** Rym Gribaa, Mehdi Slim, Chokri Kortas, Slim Kacem, Helmi Ben Salem, Sana Ouali, Elies Neffati, Fehmi Remadi, Essia Boughzela

**Affiliations:** 25 Department of Cardiology, Sahloul Hospital, Sousse, Tunisia; 26 Cardiovascular and Thoracic Surgery Department, Sahloul Hospital, Sousse, Tunisia; 27 Hôpital Sahloul, Route de la ceinture, Hammam Sousse, 4054 Sousse, Tunisia

**Keywords:** Pediatric cardiac myxoma, Pulmonary arterial obstruction, Surgery

## Abstract

**Introduction:**

Primary cardiac tumors are uncommon during infancy and childhood. Myxomas originating from the right ventricle are even less common in pediatric patients.

**Case presentation:**

Here we describe a case of an 11-year-old Tunisian boy who was referred for syncope. Transthoracic echocardiography revealed a large mobile mass attached to his right ventricle, obstructing his right ventricular outflow tract. Complete surgical excision of the mass with preservation of the pulmonary valve was performed. The diagnosis of myxoma was histologically confirmed.

**Conclusion:**

Cardiac myxomas located in the right ventricular outflow tract are rare and can present unusual diagnostic and therapeutic challenges.

## Introduction

Myxomas are the most common cardiac tumors in adults, but are rare during infancy and childhood. The prevalence of cardiac myxomas varies between 0.0017% and 0.19% in autopsy studies [1]. Only 5% of these tumors are found in the right ventricle (RV) [2]. Cardiac tumors originating from the right ventricular outflow tract (RVOT) present unusual diagnostic and therapeutic challenges. The symptoms are dependent on the size and location of the tumor. Such tumors are capable of generating major clinical consequences, including arrhythmias, pulmonary emboli, and sudden death. Once the diagnosis of the tumor obstructing the RVOT is confirmed, immediate surgery is indicated. Here we report a case of a primary cardiac myxoma located in the RVOT.

## Case presentation

An 11-year-old Tunisian boy was referred to our department for the exploration of syncope. There was no family history of myxoma. On admission, his pulse rate was 100 beats/minute and blood pressure was 100/60mmHg. He had no signs of congestive cardiac failure. On physical examination, a grade 4/6 systolic ejection murmur was heard at the left upper sternal border. His chest radiography was normal. His electrocardiogram showed normal sinus rhythm of 110 beats/minute and ST segment depression in leads V1-V4 with right ventricular hypertrophy and right axis deviation.

**Figure 1:**
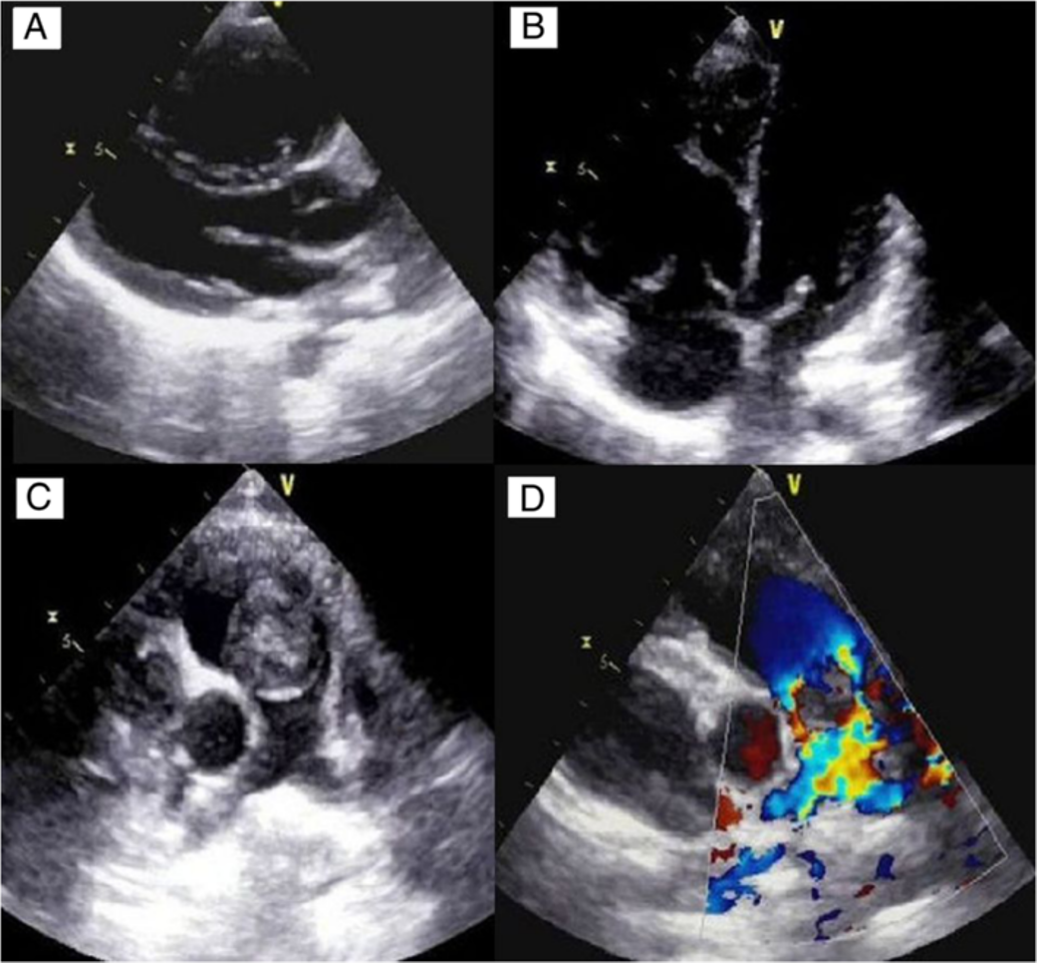
**Transthoracic echocardiography.** Parasternal long-axis view (panel **A**) and apical four chambers view (panel **B**) showing right ventricular enlargement. Parasternal short-axis view at the level of the aortic valve reveals the right ventricular outflow tract tumor (panel **C**). The obstructive character of this tumor is demonstrated by color Doppler in the same view (Panel **D**).

**Figure 2:**
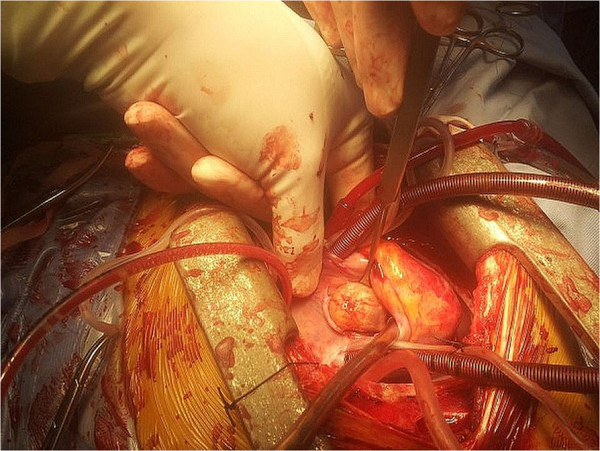
**Intraoperative view of the right ventricular myxoma.**

**Figure 3:**
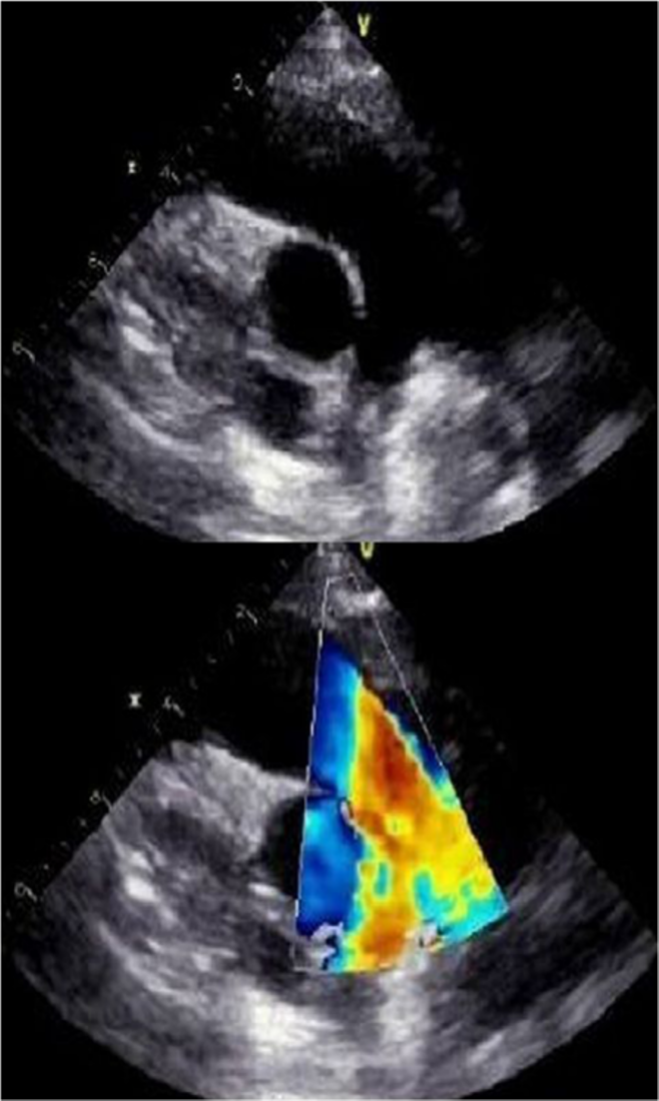
**Postoperative echocardiography.** Parasternal short-axis view at the level of the aortic valve showing an unobstructed right ventricular outflow tract.

Transthoracic echocardiography revealed a large mobile 19×19mm mass attached to RVOT. The pulmonary valve seemed to be spared. The tumor was causing an obstruction of the RVOT (Figure 1). His RV-to-pulmonary artery pressure gradient was 90mmHg. His echocardiography showed right atrium (RA) and RV dilatation, mild tricuspid regurgitation with right ventricular systolic pressure at 105mmHg. Abdominal ultrasound and venous Doppler of both his lower limbs were normal. He was anaesthetized, perioperative cardiac arrest occurred, emergency sternotomy and cardiopulmonary bypass (CPB) was established through aortic and bicaval cannulation. His RA was opened. Operative findings revealed a gelatinous 20×20mm mass, originating 1cm below the pulmonary valve and attached to the RVOT on its septal surface (Figure 2). The mass was completely removed. The diagnosis of myxoma was confirmed by histology. The postoperative period was uneventful and he was discharged after 7 postoperative days. Postoperative echocardiography documented an unobstructed RVOT (Figure 3).

## Discussion

Primary cardiac masses are rare and usually benign with myxoma being the most common [1]. Up to 75% of myxomas are located in the left atrium whereas right-sided myxomas are rare (15 to 20%), with right ventricular (3 to 4%) or pulmonary artery myxomas being extremely rare [3]. There are several case reports of right ventricular myxoma obstructing the outflow tract in adults and older children, but reports of this pathology during early childhood are scarce; cardiac myxomas in the pediatric age group have relevance in particular to a condition known as familial myxoma syndrome. This syndrome, also known as Carney’s syndrome, consists of a variable complex of mucocutaneous, visceral, and endocrine disorders. Symptoms may be variable and are determined by the tumor location and size. Right ventricular myxomas may cause obstruction of the RVOT and pulmonary main trunk, which may also lead to complications such as syncope, pulmonary embolism, and sudden death. Cardiac myxoma extending into the RVOT is a rare cause of right heart failure. Symptoms and signs include peripheral edema, ascites, and shortness of breath as a result of RVOT obstruction. The gold standard noninvasive diagnostic modality for such tumors is transthoracic or transesophageal echocardiogram. An echocardiogram enables preoperative localization of the tumor, size, shape, mobility as well as the risk of RVOT obstruction and the tumor attachment, and therefore facilitates the selection of the optimal surgical approach. Transesophageal echocardiography accurately identifies other localization of myxomas. Cardiac-gated computed tomography and cardiac-gated magnetic resonance scans offer additional information about the structure and function of cardiac tumors before surgical resection [4]. The differential diagnosis for an intracavitary cardiac mass includes thrombus, myxoma, lipoma and nonmyxomatous neoplasm, most of which are malignant [5]. The risk of life-threatening complications indicates the importance of early diagnosis and prompt surgical resection as soon as possible. One of the main concerns during anesthesia is the risk of tumor embolization and pulmonary obstruction during anesthesia induction or at any stage thereafter. Hemodynamic instability, inconvenient manipulation of the tumor and heart must be avoided to prevent these complications. Femoral arterial-venous cannulation can be performed for CPB initiation in the case of complete RVOT obstruction with hemodynamic collapse. Several surgical techniques have been suggested, but in each case it depends on the site of the tumor. Follow up for recurrent myxoma in an uncommon location is recommended.

## Conclusions

Childhood cases of right ventricular myxoma obstructing the RVOT are rare. Immediate, careful surgical resection should be performed to avoid outflow tract obstruction, which might otherwise result in sudden death.

## Consent

Written informed consent was obtained from the patient’s legal guardians/parents for publication of this case report and any accompanying images. A copy of the written consent is available for review by the Editor-in-Chief of this journal.

## Abbreviations

RV: Right ventricle
RA: Right atrium
RVOT: Right ventricular outflow tract
CPB: Cardiopulmonary bypass.

## References

[1] Gopal AS, Stathopoulos JA, Arora N, Banerjee S, Messineo F: **Differential diagnosis of intracavitary tumors obstructing the right ventricular outflow tract.** *J Am Soc Echocardiogr* 2001,**14**(9)**:**937–940. 10.1067/mje.2001.114627 10.1067/mje.2001.114627 11547282

[2] Hirota J, Akiyama K, Taniyasu N, Maisawa K, Kobayashi Y, Sakamoto N, Komatsu N: **Injury to the tricuspid valve and membranous atrioventricular septum caused by huge calcified right ventricular myxoma: report of a case.** *Circ J* 2004,**68**(8)**:**799–801. 10.1253/circj.68.799 10.1253/circj.68.799 15277742

[3] Huang SC, Lee ML, Chen SJ, Wu MZ, Chang CI: **Pulmonary artery myxoma as a rare cause of dyspnea for a young female patient.** *J Thorac Cardiovasc Surg* 2006,**131**(5)**:**1179–1180. 10.1016/j.jtcvs.2005.12.052 10.1016/j.jtcvs.2005.12.052 16678613

[4] Lacey BW, Lin A: **Radiologic evaluation of right ventricular outflow tract myxomas.** *Tex Heart Inst J* 2013,**40**(1)**:**68–70. PMC3568267 23466872

[5] Elderkin RA, Radford DJ: **Primary cardiac tumours in a paediatric population.** *J Paediatr Child Health* 2002,**38**(2)**:**173–177. 10.1046/j.1440-1754.2002.00734.x 10.1046/j.1440-1754.2002.00734.x 12031001

